# Real-Time Plane Detection with Consistency from Point Cloud Sequences

**DOI:** 10.3390/s21010140

**Published:** 2020-12-28

**Authors:** Jinxuan Xu, Qian Xie, Honghua Chen, Jun Wang

**Affiliations:** 1 College of Computer Science & Technology, Nanjing University of Aeronautics and Astronautics, Nanjing 211100, China; jinxuanxu027@gmail.com; 2 College of Mechanical & Electrical Engineering, Nanjing University of Aeronautics and Astronautics, Nanjing 211100, China; qianxie@nuaa.edu.cn (Q.X.); chenhonghuacn@gmail.com (H.C.)

**Keywords:** plane detection, point cloud sequence, depth sensor

## Abstract

Real-time consistent plane detection (RCPD) from structured point cloud sequences facilitates various high-level computer vision and robotic tasks. However, it remains a challenge. Existing techniques for plane detection suffer from a long running time or the problem that the plane detection result is not precise. Meanwhile, labels of planes are not consistent over the whole image sequence due to plane loss in the detection stage. In order to resolve these issues, we propose a novel superpixel-based real-time plane detection approach, while keeping their consistencies over frames simultaneously. In summary, our method has the following key contributions: (i) a real-time plane detection algorithm to extract planes from raw structured three-dimensional (3D) point clouds collected by depth sensors; (ii) a superpixel-based segmentation method to make the detected plane exactly match its actual boundary; and, (iii) a robust strategy to recover the missing planes by utilizing the contextual correspondences information in adjacent frames. Extensive visual and numerical experiments demonstrate that our method outperforms state-of-the-art methods in terms of efficiency and accuracy.

## 1. Introduction

Planar primitive is the most commonly-seen structure in our daily life. Thus, planar structure recognition, which can be formulated as the plane detection problem, has become an important research topic in computer vision for decades. The detected planes, which can be regarded as the abstracted form of an actual scene, contain a lot of high-level structure information and they can benefit many other semantic analysis tasks, like object detection [1], self-navigation [2], scene segmentation [3], SLAM [4,5], robot self-localization [6,7,8], For instance, the robot can better map the current environment with the plane detection result, which significantly reduces the uncertainty in the mapping results and improves the accuracy of positioning.

**Figure 1:**
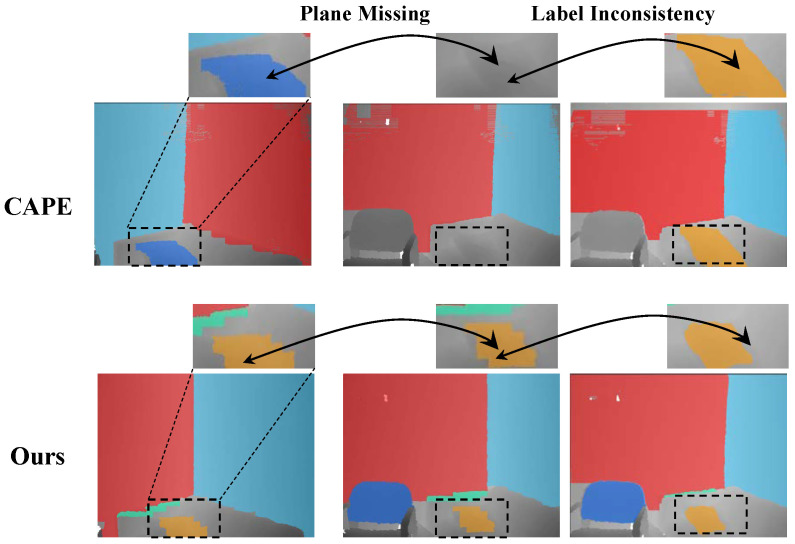
The plane flicking problem in a continuous point cloud sequence. Label inconsistency: the same plane detected from adjacent frames is labeled with different colors. Plane missing: the plane detected in the former frame is not recognized in the next frame.

Recently, RGB-D based slam [9,10,11] are emerging. Owing to this, many strategies have been proposed in order to detect planes from 3D data, like 3D point clouds, 3D mesh models, and RGB-D images. However, there are still some problems in the method of plane detection. Most of the existing plane detection algorithms are only performed on a single space [12,13,14,15]. For these methods, data are processed off-line and the relationship between frames is usually abandoned when dealing with the video input. However, consecutive plane detection in videos could also assist those algorithms that require the correspondence between frames, such as adjacent point cloud registration in SLAM [16,17]. On-line methods, like [18,19], can establish plane correspondence, but the precision of segmentation is not satisfactory. Further, the frame-by-frame strategy would cause the “flicking” problem, as shown in Figure 1. That is because planes may be lost in some frames; therefore, labels of the same plane may vary a lot. The whole issue is reflected in two aspects. First, the result of planar detection is not good at boundary segmentation or the method cannot run in real-time due to the computational overhead caused by huge data. Second, not all continuous labels are provided in the sequence of images due to plane loss in the detection stage, which has greatly limited the application of planar detection. We argue that the adjacent frames contain so much similar information that they can help each other efficiently in detecting planes. That is, the contents in adjacent frames are quite similar, since the sensor moves little during the shutter time. Thus, at the current frame, we would expect to detect those planes that are recognized in the former frame.

Therefore, we propose utilizing the superpixel segmentation in order to enhance the detection accuracy and a missing plane recovery strategy to recurrence the undetected planes. Our goal is to achieve stable plane consistency via providing more accurate plane boundary results and stable plane sequences. We only take the raw depth information as input in order to reduce the time consumption and the color shade that are caused by the illumination variation. Additionally, 3D points with their surroundings are more robust during inferring planar structures, which are point-wise semantic information.

Our method can be summarized into two main stages: plane detection and plane correspondence establishment. Both of these two stages are performed in real-time, which means that our method can be performed online. In the plane detection stage, we propose employing the super-pixel segmentation as the pre-process to achieve more smooth and accurate plane boundary.

We then introduce a plane matching strategy to establish correspondences between the detected planes in two adjacent frames. Note that the whole algorithm is performed in real time. Therefore, it could be applied to many online vision tasks, such as real-time 3D reconstruction [9,10,20,21,22].

Overall, our contributions are as follows:

- We introduce a real-time plane extraction algorithm from consecutive raw 3D point clouds collected by RGB-D sensors.
- We propose a superpixel-based plane detection method in order to achieve smooth and accurate plane boundary.
- We present a strategy for the recovery of undetected planes by utilizing the information from the corresponding planes in adjacent frames.

The rest of this paper is organized, as follows. Section 2 gives a brief review of the related works. Section 3 presents the overview and a full description of the proposed algorithm, including the details of the plane detection method in a single frame and plane tracking across frames. Section 4 presents the experimental evaluation results. Finally, Section 5 discusses the conclusion and future work.

## 2. Related Work

### 2.1. Patch Segmentation

Superpixel segmentation trying to partition a still image into atomic segments of similar size and adhering to object boundaries was first introduced for image analysis and processing by Ren and Malik [23]. With the advent of 3D laser data, many superpixel segmentation methods have also migrated naturally to 3D data processing. Moore et al. [24], Veksler et al. [25], Weikersdorfer et al. [26], Zhou et al. [27], and Picciau et al. [28] give their solution for both 2D superpixels and 3D supervoxels. However, their method is on the premise that the data are uniformly distributed.

Song et al. [29] propose a two-stage method in order to make the algorithm more adaptable and to make the supervoxels conform better to object boundaries. In the first stage, boundary points are estimated by the discontinuity of consecutive points along the scan line. In the second stage, a neighborhood graph that excludes the edges that are connected by boundary points is constructed, and a clustering process is performed on the graph to segment the point clouds into supervoxels.

Lin [30,31] reformulates the K-means problem over supervoxels into a subset selection problem and develops a heuristic algorithm that utilizes local information to efficiently solve the subset selection problem. This method can produce supervoxels with adaptive resolutions, and it does not rely on the selection of seed points.

### 2.2. Plane Detection

The problem of plane detection has been researched for many years. With the many methods that have been proposed, most of them fall into three categories: Random Sample Consensus-based (RANSAC-based), Hough Transform-based, and Region Growing-based methods.

**RANSAC-based.** RANSAC is a non-deterministic method for parameterized object adjustment. Given a certain degree of confidence, the algorithm randomly selects data points to fit the primitives iteratively and picks the best model under the specific rule. Thanks to its robustness on noise and outliers data, a considerable number of RANSAC based plane detection methods [32,33,34] have been proposed. Bostanci et al. [35] proposed a sequential RANSAC algorithm in order to find planar features from 3D point clouds captured by Kinect. The algorithm reduces storage costs, by using the explicit definition of the plane that allows for storing only four parameters per plane rather than thousands of points.

Parallelism is leveraged to speed up the RANSAC algorithm, reaching an average speed up to twice the ratio. Biswas et al. [36] introduced the local RANSAC over the constrained window region and applied it to indoor mobile robot localization and navigation. Lee et al. [37] estimated local surface normal and curvature by a simple spherical model, and then segment points while using a modified flood fill algorithm. After an initialization plane model using RANSAC, they carry out a model refinement and boundary extraction step in order to refine the result. However, only one primitive can be extracted at each execution due to the intrinsic mechanism of the RANSAC algorithm.

**Hough transform-based.** Hough transform [38] is another widely used method for detecting parameterized objects. For any given input, it casts the data from the coordinate space to the parameter space and executes a point-by-point vote for each latent primitive. The votes of all data points are accumulated and primitives with the most votes are chosen as potential primitives. Works that are based on Hough transform mainly include [14,39,40,41]. Nguyen et al. [40] proposed a Hough transform-based method to accurately detect planes. They first estimate surface normal vectors of points and define the plane orientations, by mapping the estimated normals to a Gaussian map, called IJK space. Clustering and further optimization are then conducted, taking the normal distribution in IJK space as input. Borrmann et al. [42] analyzed the performance of different Hough methods and propose an improved accumulator for plane detection that improves the original performance. Limberger et al. [43] proposed a real-time detection method of the planar region that is based on an efficient Hough transform voting scheme, by clustering approximately co-planar points and by casting votes for these clusters on a spherical accumulator while using a trivariate Gaussian kernel. Vera et al. [14] presented a real-time Hough transform method while using an implicit Quad-tree to identify clusters of approximately co-planar points in the 2.5-D space. The detection is performed while using an efficient Hough transform voting scheme that models the uncertainty that is associated with the best-fitting plane for each cluster as a trivariate-Gaussian distribution.

**Region growing based.** In contrast to the above-mentioned approaches, region growing based algorithms take advantage of the connectivity information and then group data according to specific properties. For instance, in [44], a two-stage method is proposed, which consists of a plane fitting and a polygonalization step. Utilizing the sequential nature of 3D data that are acquired from mobile robots, a point-wise region growing operation is conducted in order to iteratively collect plane points, with each time adding one point into the plane groups. The growing criterion is whether the mean square error (MSE) is lower than the given threshold. For rapid detection, techniques, like priority search queue, are used, although the execution time is still not satisfactory. This is because an eigen decomposition step is involved, in addition to the nearest neighbor search. In order to reduce the cost to merge planes, Holz et al. [45] instead suggests updating the plane parameters employing approximate polygonal meshing. This requires point normal computation, which is known to be costly. Utilizing the image structure of the range map, Holz et al. [46] proposes an efficient solution, by using integral images in order to compute the local surface normal. Trevor et al. [47] also introduce a connected component-based approach to perform segmentation that operates on organized point cloud data and achieves real-time performance (30 Hz). While the above-mentioned methods make attempts to detect planes that are based on the region growing algorithm, the computational cost can be significantly reduced by cluster (grids) level operation, instead of operating on a single data point. Feng et al. [12] proposed a real-time plane detection method for range image that was obtained from Kinect-liked sensors. With the pre-processing uniformly dividing the range map into non-overlapping grids, a graph is constructed, in which the node and edge represent the point and neighborhood information. Agglomerative hierarchical clustering is employed in order to merge points that belong to the same plane. Finally, a pixel-wise region growing is executed to refine the extracted plane. Based on [12], a new method is proposed by Proena et al. [19] for distinguishing cylindrical surfaces from the detected planes. When compared with [12], the latency of [19] is more consistent and 4–10 times faster, depending on the scene. In [12,19], the pre-process is the key to making the algorithm run in real-time, which divides the range map into grid specific resolution, and it keeps a grid-level operation in most subsequent steps. This pre-process undoubtedly reduces the operation time in practice.

However, the imprecise partition, which segments the original image into square patches, makes the boundary of the segments diverge from the actual plane boundary, resulting in a vague plane detection result. Our method follows this approach of conducting super-pixel wise operation. However, our method gives a more precise real-time segmentation result, by performing a constrained K-means clustering at the beginning. For point cloud sequences, we establish an inter-frame plane corresponding relationship besides the intra-frame plane detection. We also design an efficient strategy to recover an undetected plane, due to sensor instability.

## 3. Method

**Figure 2:**
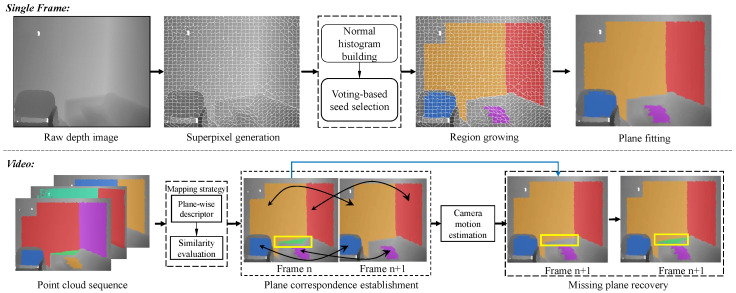
Pipeline of the proposed plane detection method on a given depth video. Note that our method does not rely on any color information.

Our algorithm expects a continuous structured point cloud sequence that is captured by RGB-D sensors as input and aims at detecting consistent plane structures over all frames in real time. Figure 2 summarizes the pipeline of our method. Please note that we also name the structured point cloud an image frame, due to the regular format of RGB-D data.

Our algorithm generally contains two steps: extracting all of the reliable planes for each frame and building frame-to-frame plane correspondences in the whole sequence. Specifically, we start by generating edge-aware superpixels and then distinguish the superpixel-wise planar and non-planar regions. Furthermore, all of the reliable planar structures are extracted in each image in Section 3.1. In the subsequent frame-to-frame step, the one-to-one plane correspondences are established based on a 6D descriptor in Section 3.2. Ultimately, missing planes are recurred by the proposed plane recovery strategy that is described in Section 3.3.

### 3.1. Plane Detection in Single Frame

In this section, we explain how to identify all the reliable plane structures in each image (namely structured point cloud). In order to guarantee the plane detection running in real-time and ensure that the plane structures are reliable, our method first generates edge-aware superpixels rapidly, followed by planar superpixel identification and merging, and all reliable planes finally are fitted.

**Figure 3:**
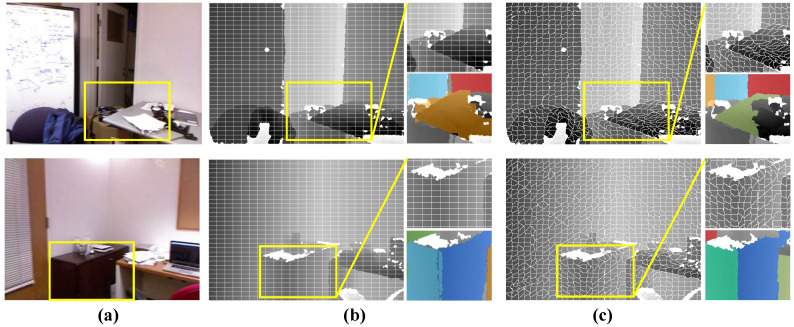
Comparison of the superpixels generated by different segmentation schemes. From left column to right column: (**a**) the real scene image, (**b**) the superpixel results of [19], and (**c**) our method. We can easily observe that our method produces edge-aware superpixels, which provide a better basis for later plane extraction (see the bottom-right plane fitting results).

**Edge-aware Superpixel Generation.** Dividing the input image into superpixels is an essential step to keep the full algorithm running in real-time [19]. Proença et al. [19] used the simplest method: regularly segmenting the image into grids of a specific resolution. Although it can significantly shorten the processing time, the plane detection accuracy will be affected, especially for some challenging regions, where the segmented grid borders are difficult to exactly match the prominent image structures (see Figure 3b). In order to solve this problem, we employ an improved K-means based clustering scheme to generate edge-aware superpixels with nearly equal size, whose borders comply well with the real plane edges and, thus, produce a more precise detection result, as illustrated in Figure 3c. When compared with the method that performs supervoxel segmentation directly on the point cloud, like [30,31], there are similarities and differences in our algorithm. Both the work of Lin et al. [30,31] and ours take advantage of local k-means clustering in order to accelerate the algorithms, the main difference is that our approach directly exploits the structure of the image. Consequently, all of the points in the same superpixel block are next to each other in the image. This allows for the subsequent cross-check steps to coarsely remove non-planar parts and further reduce the size of the problem.

The traditional K-means technique requires each pixel to engage in the calculation of each superpixel, and the clustering procedure is time-consuming. The size of the superpixels may also vary considerably, which will lead the larger superpixels to be more likely regarded as a planar region in follow-up steps. Hence, we strictly restrain the search region of each superpixel. The search region is set as $2S_{x}\times 2S_{y}$ around each seed pixel, where $S_{x}=N_{x}/k$, $S_{y}=N_{y}/k$, $N_{x}$, and $N_{y}$ are the width and height of the image, and *k* is a parameter representing the desired grid number. We set the seed pixel at the positions with the lowest gradient within a 3×3 neighborhood in order to encourage the attention of superpixels less on the edge regions. Meanwhile, using the distance metric in image space or 3D space cannot achieve an accurate clustering result. Some pixels from different objects may be clustered into one superpixel, since there often exists large depth variations among neighboring pixels. In order to solve this issue, we define a new bounded metric *D*, as follows:

(1) $$D=\sqrt{{\left(\frac{d_{xy}}{R_{xy}}\right)}^{2}+{\left(\frac{d_{depth}}{R_{depth}}\right)}^{2}}$$

where $d_{xy}$ is the distance in image space, $d_{depth}$ is the distance in depth space, $R_{xy}$ is the upper bound of the K-means search range in image space, and $R_{depth}$ is the scale factor in depth space. We can set different combinations of $R_{xy}$ and $R_{depth}$ to balance the effects of the two metric components. In our experiment, we set $R_{xy}=\sqrt{S_{x}^{2}+S_{y}^{2}}$ to normalize to the distance in image space, while $R_{depth}$ is tunable according to the input data. Note that the RGB color information can also be incorporated into the metric in order to promote the effect, but it is not suitable for texture-less objects. Hence, we only use the geometric information, but users can freely add any extra information.

The complete process of superpixels segmentation is as follows: first, initialize cluster center $C_{k}$ by sampling pixels at regular grid steps S. Subsequently, move cluster centers to the lowest gradient position in a 3×3 neighborhood. For each cluster center $C_{k}$, the distance D for each pixel *p* within its search region is computed. The pixels *p* are assigned to the cluster with the minimum distance D.

**Planar Superpixels Identification.** After subdividing each image into small edge-aware superpixels, we need to identify all of the flat superpixels and merge them to form complete plane structures. The plane discrimination mainly comes from [19]. We reiterate this in conjunction with the previous superpixel step. We adopt the straightforward “cross-check” to check obvious non-planar superpixels. Specifically, for each superpixel $C_{i}$, when the neighboring pixels around its center have a depth difference larger than a specific value $c_{0}$, the current superpixel will be considered to be a non-planar structure. After the above coarse detection, those potential plane superpixels will undergo a fine check. Given a potential plane superpixel $C_{i}$, the principal component analysis (PCA) is performed first. We can obtain the smallest eigenvalue $\lambda_{i}^{3}$ and its corresponding eigenvector $n_{i}$, which can be regarded as $C_{i}$’s normal vector. If $\lambda_{i}^{3}$ is less than ${\left(\sigma_{z}+\epsilon \right)}^{2}$, then $C_{i}$ will be labeled as a flat area. $\sigma_{z}$ is the sensor uncertainty and ϵ is the tolerance coefficient. Both of them are hardware-related. In our cases, $\sigma_{z}$ is equal to $1.425*10^{-6}*{\left(\lambda_{i}^{3}\right)}^{2}$, and ϵ is set as 15.

**Planar Superpixels Merging.** In this stage, all of the planar superpixels belonging to the same plane structure are collected to form the final complete smooth plane, via a superpixel-wise region growing scheme. To this end, we first build a normal histogram according to the normal angles of superpixels in the spherical coordinate system. Specifically, the polar angles and the azimuth are uniformly divided, according to the quantization step. Normal vectors falling into the same region are assigned to the same bin. During each iteration of the region search, the initial seed is selected from the bin with the most votes. The normal histogram is dynamically updated among the unassigned superpixels after each iteration.

When searching the similar superpixels from the seed superpixel $C_{t}$, the neighboring superpixel $C_{i}$ will be labeled in the same plane region with $C_{t}$, only if it meets the following conditions:

- $C_{i}$ is unlabeled;
- the normal angle difference between $n_{i}$ and $n_{t}$ is less than a given threshold θ, which is set as $15^{\circ }$ by default in our experiments; and,
- the distance from $C_{i}$’s centroid $m_{i}$ to the $C_{t}$’s fitting plane is less than $Td\left(m_{i}\right)=l*\sqrt{N_{i}}*\sin\theta$, where $N_{i}$ is the total number of 3D points in the current merged superpixels and *l* is the merge distance threshold.

**Figure 4:**
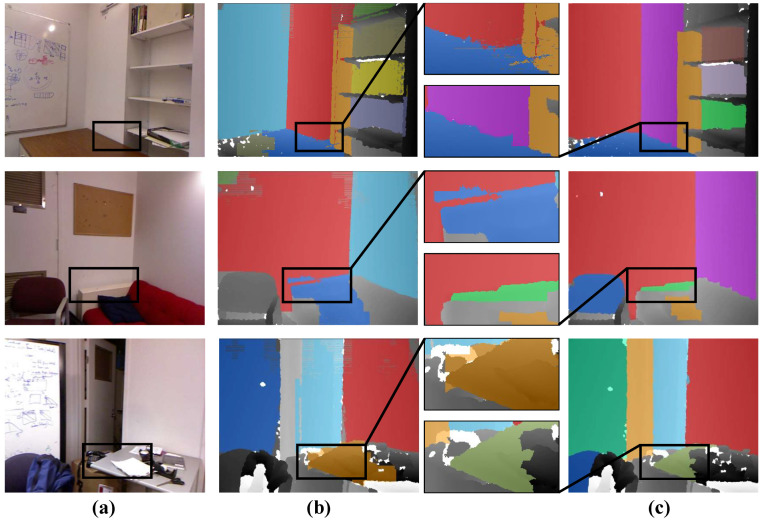
Plane detection results on the single frames. From left column to right column: (**a**) the real scene image, (**b**) the result of CAPE [19], and (**c**) our method. As shown, our method correctly detects more planes and performs better in terms of accuracy.

It is worth noting that the distance threshold $Td(m_{i})$ is adaptive with $N_{i}$ and θ. This is, because, in some practical cases, the large plane contains a larger depth range. Finally, the complete plane structure can be fitted, based on the merged planar superpixels. Our approach detects more planes, while keeping the plane structure relatively high quality, especially in the edge regions, as shown in Figure 4.

### 3.2. Plane Correspondence Establishment

In order to establish plane correspondences among frames, we introduce a six-dimensional (6D) descriptor based on the observation that the camera poses of adjacent frames change slightly. For the plane $P_{i}^{f}$ in frame *f*, the descriptor is defined, as follows:

(2) $$d_{P_{i}^{f}}=\left[\bar{X_{P_{i}^{f}}},\bar{Y_{P_{i}^{f}}},\bar{depth_{P_{i}^{f}}},n_{P_{i}^{f}}^{x},n_{P_{i}^{f}}^{y},n_{P_{i}^{f}}^{z}\right]$$

The first three components are the 3D coordinates of $P_{i}^{f}$’s centroid, and the latter are the normal vector.

**Figure 5:**
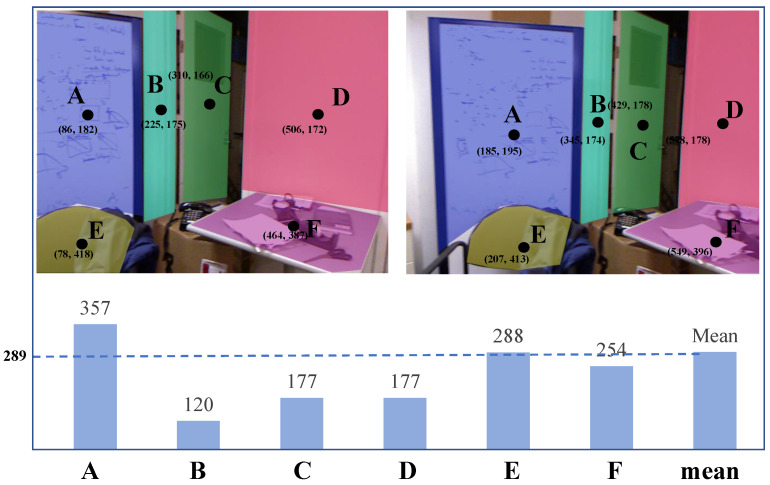
Illustration of plane centroid drift. As the camera moves by a small angle, the centroid of the detected planes may obviously change, due to the large distance variation in the depth direction.

Although the camera pose difference may be small in adjacent frames in general, sometimes the sensor suffers from a missing frame. In other words, frames may be lost during the process of data transmission of the sensors. As a result, the visible part of some objects in adjacent images sometimes varies drastically, which causes a large motion of the centroid of the detected plane (e.g., the whole structure of one object gradually appears in the image as the camera moves, and the centroid position of the plane fluctuates greatly), as shown in Figure 5. In order to enhance the performance of our descriptor in this situation, we reformulate it as:

(3) $$d_{P_{i}^{f}}^{\prime }=\left[\bar{X_{P_{i}^{f}}}/N_{P_{i}^{f}},\bar{Y_{P_{i}^{f}}}/N_{P_{i}^{f}},\bar{depth_{P_{i}^{f}}}/N_{P_{i}^{f}},n_{P_{i}^{f}}^{x},n_{P_{i}^{f}}^{y},n_{P_{i}^{f}}^{z}\right]$$

where $N_{P_{i}}^{f}$ is the total number of pixels of the plane $P_{i}^{f}$. We then build the plane correspondences by computing the Euclidean distance of the descriptors. In the adjacent frames, planes $P_{j}^{m}$ and $P_{j+1}^{n}$ from frames $F_{j}$ and $F_{j+1}$ are assigned the same label, if and only if they meet the following conditions:

- the Euclidean distance of descriptors $d_{P_{j}^{m}}^{\prime }$ and $d_{P_{j+1}^{n}}^{\prime }$ is smaller than the given threshold $d_{0}$;
- there are no other planes in Frame $F_{j+1}$ whose descriptor is closer to the plane $d_{P_{j}^{m}}^{\prime }$; and,
- if descriptor of plane $P_{j+1}^{n}$ is the smallest one to more than one plane in Frame $F_{j+1}$, $P_{j+1}^{n}$ would be assigned the label of the plane whose descriptor is the closest to it.

### 3.3. Undetected Plane Recovery

Up to now, our method can detect all of the reliable consistent plane structures over frames. However, in certain cases, some planes cannot be detected. Based on the assumption that the transformation between adjacent frames is linear, in this section we propose utilizing the contextual information in order to estimate the camera motion trajectory, thus restoring the missing planes and their correspondences with planes in adjacent frames.

Assuming that there exists a plane $P_{o}^{f+1}$ not detected in frame f+1, but its corresponding plane $P_{o}^{f}$ in frame *f* was detected. We first compute a translation *T*, according to the identified paired planes:

(4) $$T=\frac{\sum (m_{P_{i}^{f+1}}-m_{P_{i}^{f}})}{N(P_{i})}$$

where $P_{i}^{f+1}$ and $P_{i}^{f}$ are the corresponding planes that are acquired through previous steps, and $m_{P_{i}^{f+1}}$ and $m_{P_{i}^{f}}$ are their centroids. We then judge whether the centroid of $P_{o}^{t}$ moves out of the image range in the f+1 frame. This can be easily obtained by:

(5) $$m_{P_{o}^{f+1}}=\left(m_{p_{o}^{f}}+T\right)*K^{-1}$$

where *K* is the internal matrix of the sensor. If the estimated $m_{P_{o}^{f+1}}$ locates in the image range, we relax the plane determination condition and relaunch the region growing step by five percent each time, until the missing planes are recovered. If the region growing iteration is 1.5 times, then the plane is judged to disappear.

## 4. Experiments and Results

This section provides experimental results on raw RGB-D benchmark datasets to validate the performance of our algorithm. The experiments are conducted on scenes of single- and multi-frame cases. Note that our method only takes the depth information as input, while the RGB information is not used.

**Parameter Setting.**$N_{x}$: the number of superpixels of an image divided in the X direction, is fixed to 20 for both datsets, $N_{y}$: the number of superpixels of an image divided in the Y direction, is fixed to 15 for both datsets, $c_{0}$: the maximum difference between adjacent pixels, is fixed to 100 for the NYU dataset and to 4 for the SR400 dataset [15], *l*: the threshold for planar region merging, is fixed to 1000 for the NYU dataset and 36.5 for the SR400 dataset [15], and *E*: threshold in plane correpsondence discrimination, is fixed to 0.0115.

**Evaluation Dataset.** The proposed algorithm is evaluated on the NYU dataset [48] and the SR4000 dataset [15], where most planar content will be encountered in real life. The NYU dataset [48] is captured by Kinect with a resolution of 640 × 480 pixels. Without any further processing, the proposed methods can be tested on the raw depth map with optical distortions that are introduced by the device directly. The SR4000 dataset [15] contains depth images which are generated by the ToF depth camera. This dataset presents typical indoor scenes and the pixel resolution is 176 × 144. The pixel-level ground truth is labeled manually.

**Competitors.** We compare our method with several state-of-the-art plane detection methods [12,15,18,19]. In order to evaluate the effectiveness of our method on both single depth image and multi-frame depth videos, we first compare our method with the on-line method from Proena et al. [19] and off-line methods from Jin et al. [15] and Feng et al. [12] on the single-frame data that are based on the above dataset. For the sake of clarification, we refer to the method in [19] as CAPE and the method in [15] as DPD. For multi-frame data, we compare with on-line method CAPE [19] and compare with on-line methods CAPE [19] and CAPE+, which is a combination of plane detection method in [19] and the plane matching strategy in [18]. Note that CAPE+ establishes plane relationships that are based on mini-mask overlaps, angle of normal of plane, and plane-to-plane distance. We carefully tune all of the parameters of the competitors to achieve the best results.

### 4.1. Experiment #1: Plane Detection in Single Frame

In this section, we evaluate the effectiveness and efficiency of the proposed method for extracting planes in single-frame cases.

**Figure 6:**
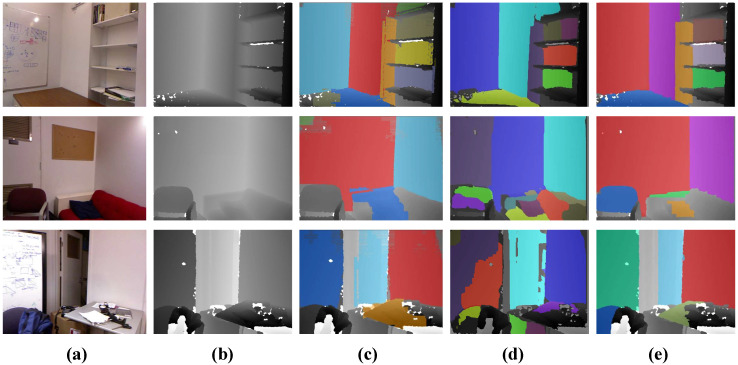
Plane detection results on the NYU dataset. From left column to right column: (**a**) the real scene image, (**b**) the raw depth map, (**c**) the result of CAPE [19], (**d**) the result of [12] and (**e**) ours. The result of CAPE [19] is unsatisfactory in the boundary regions, while the method of Feng et al. [12] often over-segments some complete planes.

**Figure 7:**
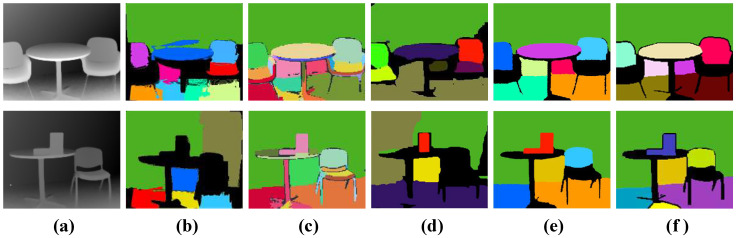
Single-frame result on the SR4000 dataset [15]. (**a**) the input depth map, (**b**) the result of CAPE [19], (**c**) DPD [15], (**d**) Feng et al. [12], (**e**) ours, (**f**) the corresponding ground truth.

Figure 6 gives the visual results on several frames from the NYU dataset. We can observe that the results of our method are substantially better than CAPE [19] and Feng et al. [12]. Taking the third row in Figure 6 as an example, when the input is a cluttered desktop, our method can effectively detect this reliable desktop plane with its edges exactly matching the image structures. The method that was proposed by Feng et al. [12] can also detect most potential planes, but it easily leads to the over-segmentation of a complete plane structure (see the second row, Figure 6d). Note that we plot the detected plane structures in random colors for better visualization. The additional visual comparisons are also conducted on the SR4000 dataset [15]. Figure 7 demonstrates that our approach is capable of correctly detecting all of the planes. Even compared with off-lined methods [12,15], our method still performs better.

Apart from visual comparison, we quantitatively assess the plane extraction results of the approaches that are involved by three metrics: (1) detection sensitivity (SE), which can be computed by SE=TP/(TP+FN); (2) detection specificity (SP), which is computed by SP=TN/(TN+FP); and, (3) correct detection ratio (CDR), which counts the labeled planes that have been successfully detected as inliners of the plane, and one plane that has over 80% overlap with the ground truth is regarded as a correct detected plane. This is also the metricthat is used in DPD [15]. In order to keep the comparison fair, we continue to use this method of comparison in this article. TN (True negative) counts the non-belonging pixels that have been successfully detected as outliers of the plane. FN (False negative) and FP (False positive) count the pixels that are wrongly classified as not belonging and belonging to the plane, respectively. Table 1 shows the quantitative results of the competitors and ours. When compared with on-line method CAPE [19], the performance of ours is much better. Even compared with off-line methods, which have a computation of orders of magnitude larger than ours, our result is still comparable.

Table 2 provides the running time of the methods. As shown, our method is much faster than DPD [15] and Feng et al. [12]. Although it is a little slower than CAPE [19], it is still acceptable for real-time performance. Note that, in the experiment, DPD [15] takes more than 7 min to generate one frame result. We think, with such a big difference in running time, that it makes no sense to compare the results of these experiments. Therefore, the result is not listed in Table 2.

### 4.2. Experiment #2: Plane Detection in Frame Sequences

**Figure 8:**
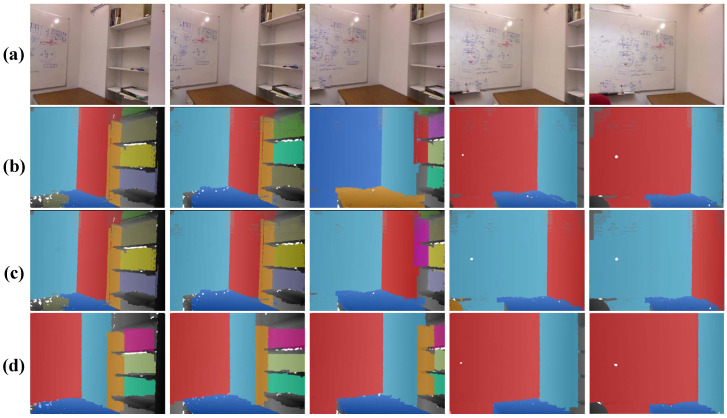
Multi-frame detection result on the NYU dataset [48]. From top row to bottom: (**a**) the real scene image, (**b**) the result of CAPE [19], (**c**) the result of CAPE+ [18], and (**d**) ours.

In this section, we evaluate our method on five successive scenes over 300 frames in the NYU dataset [48]. The results on several frames are demonstrated in Figure 8. It is easy to observe that results of the CAPE [19] flick greatly, since this method has no mechanism to recover plane correspondence relationships over continuous frames.

CAPE+ is able to keep the correspondences for most planes, but the problem of the label mismatching still occured, due to the lack of missing plane recovery strategy. By contrast, our method yields consistent plane labels over frames. Furthermore, in order to quantitatively assess the performance of our method on continuous input data, we conduct our method on three real scenes, which totally contain over 150 image frames. Table 3 reports the evaluation results.

We take PFF and PMF as the evaluation metrics: PFF (plane flicking frequency) counts the plane flicking times in all image sequences and PMF (plane missing frequency) counts the frequency that planes are not detected in all of the image sequences.

From Table 3, we can observe that our method outperforms CAPE [19] and CAPE+ [18], on all three scenes. We can also find that PFF of CAPE+ [18] decreases obviously when compared with CAPE [19], since CAPE+ also uses a plane matching strategy. However, the PFF and PMF of CAPE+ are still higher than our method due to lack of missing plane recovery scheme. In general, our method works better, because our method includes both the plane correspondence establishment step and the missing plane recovery step.

In order to analyze the pros and cons of the two methods objectively, we conduct another set of experiments taking the plane detection result of ours as input for plane matching stage. For CAPE+, the plane mini-mask overlap rate is set to 50%, which is also used in [18]. The result is listed in Table 4. As can be seen, the plane matching result are quite similar. That is because the mini-mask overlap rate of the same plane is high; as a result, the overlapping item distinguish rare planes and the other criteria of the two strategies are functionally equivalent.

### 4.3. Experiment #3: Ablative Analysis

**Figure 9:**
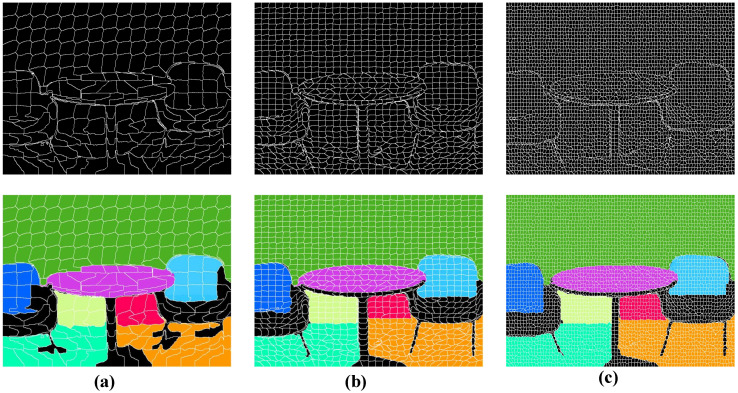
Comparing the plane detection results by different superpixel sizes: (**a**) 40 × 36, (**b**) 20 × 15, and (**c**) 8 × 10. The first row shows the superpixel segmentation results, and the second row is the corresponding plane detection results.

**Figure 10:**
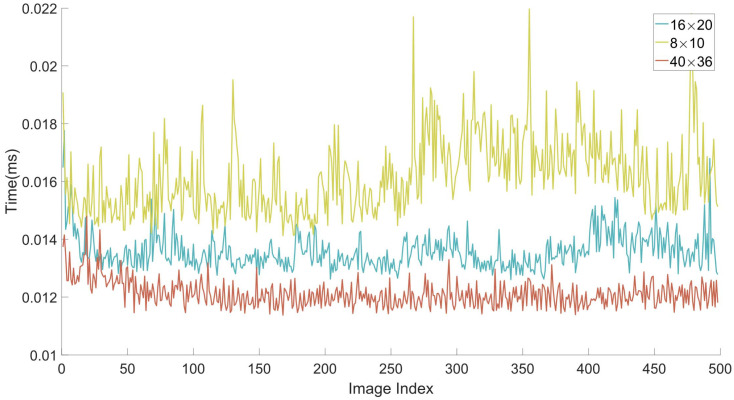
The running time under different superpixel size.

In order to analyze the contribution of major parameters or components in our method to the final performance, including superpixel size (in Section 3.1) and missing plane recovery (in Section 3.3), we conduct an ablation study in this section. We first carried out experiments under different superpixel size. The quantity analysis and visual effect of plane quality are both given (Figure 9 and Table 5). Furthermore, the runtime under different conditions is shown in Figure 10. By comparing the segmentation results, it can be noticed that employing smaller superpixel size can achieve more accurate segmentation result, both in the superpixel segmentation stage and the final plane detection stage (Figure 9). However, it consumes more time to generate superpixels (Figure 10) at the same time.

**Figure 11:**
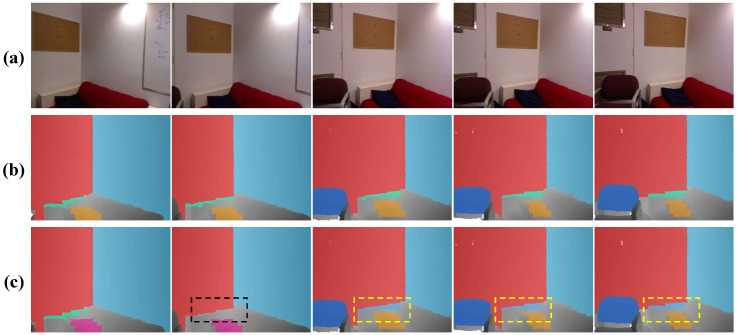
Comparison of the proposed method with and without the plane recovery step. From top row to bottom: (**a**) the real scene, (**b**) the results with plane recovery, and (**c**) the result without plane recovery. The black dashed box represents an undetected plane, and the yellow dash boxes indicate the planes, whose labels are inconsistent with the plane in the first frame.

Figure 11 shows the comparison of the proposed method with and without the plane recovery step. As can be seen, the one without a plane recovery step suffers from missing planes and label inconsistency.

### 4.4. Limitation

**Figure 12:**
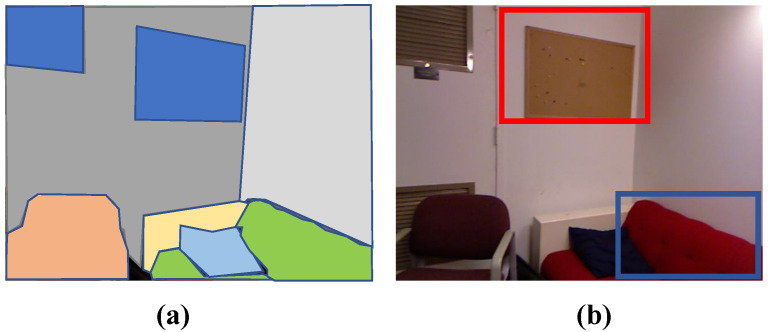
Limitations. (**a**) a manually labeled segmentation result, (**b**) the corresponding real scene. Red box indicates the plane with shallow depth variance against its surrounding plane. Blue box indicates other primitive type, i.e., cylinder.

Figure 12 illustrates two limitations of our method. One of them is the ability to distinguish thin planes, due to shallow value variation in the depth direction. Secondly, non-planar primitive detection is not implemented in this work, which is our future work.

## 5. Conclusions

In this work, we try to solve the challenging problem of real-time consistent plane detection from the raw point cloud sequence that is captured by depth sensors. We first propose detecting all reliable plane structures in a single frame. An effective mechanism is then introduced in order to establish the one-to-one plane correspondences over frames. Finally, we present a plane recovery strategy to re-identify those missing planes that are caused by sensor jitter. Extensive experiments demonstrate that our method achieves a comparable plane detection result with off-line methods in single-frame cases, while it outperforms one-line methods in multi-frame cases.

## Figures and Tables

**Table 1:** Quantitative comparison on the SR4000 dataset (%). The optimal result is bolded.

Methods		CAPE [19]	DPD [15]	Feng et al. [12]	Ours
	SE	65.62	**91.89**	71.40	90.57
Scene1	SP	89.55	93.83	**94.85**	92.98
	CDR	62.50	**100.00**	75.00	**100.00**
	SE	55.86	**93.81**	68.52	90.89
Scene2	SP	93.45	**97.58**	94.85	96.83
	CDR	33.33	**100.00**	66.67	**100.00**

**Table 2:** Average runtime of methods on the NYU dataset and the SR4000 dataset. The optimal result is bolded.

Data Sets	CAPE [19]	DPD [15]	Feng et al. [12]	Ours
NYU dataset	**3 ms**	**-**	7000 ms	14 ms
SR4000 dataset	**1 ms**	43.17 s	532 ms	2 ms

**Table 3:** Plane label flick frequency (PFF) and plane missing frequency (PMF) of CAPE [19] and our method in Figure 8. The smaller the value, the better the performance. The optimal result is bolded.

	Total Planes	CAPE [19]		CAPE+ [18]		Ours	
		PFF	PMF	PFF	PMF	PFF	PMF
scene 1	350	186	23	25	23	**7**	**5**
scene 2	165	43	5	5	5	**0**	**0**
scene 3	276	122	17	18	17	**3**	**2**

**Table 4:** The PFF and the PMF of CAPE+ and ours with the same input.

	Total Planes	CAPE+		Ours	
		PFF	PMF	PFF	PMF
scene 1	350	9	5	7	5
scene 2	165	0	0	0	0
scene 3	276	2	2	3	2

**Table 5:** Time statistics of segmentation step with different superpixel sizes.

Superpixel Size	SE	SP
8 × 10	91.33	94.17
20 × 15	90.57	92.98
40 × 36	88.26	90.44

## Data Availability

Not applicable.
